# Structural effects of inosine substitution in telomeric DNA quadruplex

**DOI:** 10.3389/fchem.2024.1330378

**Published:** 2024-01-19

**Authors:** Ya Ying Zheng, Ricky Dartawan, Yuhan Wu, Chengze Wu, Hope Zhang, Jeanne Lu, Ashley Hu, Sweta Vangaveti, Jia Sheng

**Affiliations:** ^1^ Department of Chemistry, Albany, NY, United States; ^2^ The RNA Institute, University at Albany, State University of New York, Albany, NY, United States

**Keywords:** G-quadruplex, telomeric repeats, inosine, G-I mutation, CD spectra

## Abstract

The telomeric DNA, a distal region of eukaryotic chromosome containing guanine-rich repetitive sequence of (TTAGGG)n, has been shown to adopt higher-order structures, specifically G-quadruplexes (G4s). Previous studies have demonstrated the implication of G4 in tumor inhibition through chromosome maintenance and manipulation of oncogene expression featuring their G-rich promoter regions. Besides higher order structures, several regulatory roles are attributed to DNA epigenetic markers. In this work, we investigated how the structural dynamics of a G-quadruplex, formed by the telomeric sequence, is affected by inosine, a prevalent modified nucleotide. We used the standard (TTAGGG)_n_ telomere repeats with guanosine mutated to inosine at each G position. Sequences (GGG)_4_, (IGG)_4_, (GIG)_4_, (GGI)_4_, (IGI)_4_, (IIG)_4_, (GII)_4,_ and (III)_4_, bridged by TTA linker, are studied using biophysical experiments and molecular modeling. The effects of metal cations in quadruplex folding were explored in both Na^+^ and K^+^ containing buffers using CD and UV-melting studies. Our results show that antiparallel quadruplex topology forms with the native sequence (GGG)_4_ and the terminal modified DNAs (IGG)_4_ and (GGI)_4_ in both Na^+^ and K^+^ containing buffers. Specifically, quadruplex hybrid was observed for (GGG)_4_ in K^+^ buffer. Among the other modified sequences, (GIG)_4_, (IGI)_4_ and (GII)_4_ show parallel features, while (IIG)_4_ and (III)_4_ show no detectable conformation in the presence of either Na^+^ or K^+^. Our studies indicate that terminal lesions (IGG)_4_ and (GGI)_4_ may induce certain unknown conformations. The folding dynamics become undetectable in the presence of more than one inosine substitution except (IGI)_4_ in both buffer ions. In addition, both UV melting and CD melting studies implied that in most cases the K^+^ cation confers more thermodynamic stability compared to Na^+^. Collectively, our conformational studies revealed the diverse structural polymorphisms of G4 with position dependent G-to-I mutations in different ion conditions.

## Introduction

The postulated Watson-Crick model of DNA has revolutionized the study in genetics and underpinned the existing understanding of heritability in living organisms at a molecular level. The double helical structure of DNA encodes genetic information via complementarity with G:C and A:T pairs. This eminent double helical framework provided insights into both the accessibility and packaging for genetic materials. The precise sequence of bases dictates the instructional properties for downstream transcription and translation (Travers and Muskhelishvili, 2015). However the discovery of non-helical DNA motifs and DNA modifications, and their critical roles in regulating gene expression (Liyanage et al., 2014), implies that DNA plays more than just the passive role of housing the genetic repertoire. While epigenetic markers control chromatin remodeling and transcription, telomeres at the distal region of the eukaryotic chromosome are one of the key factors in cell senescence where high levels of telomerase activity is associated with tumorigenicity (Harley et al., 1990; Bodnar et al., 1998; Hahn et al., 1999; Mergny et al., 2002).

The telomeric regions consist of guanine-rich tandem repeats of (TTAGGG)n that can adopt a quadruplex structure with thymine and adenine in the connecting loops (Wellinger and Sen, 1997; Bryan, 2020). These guanine-rich motifs are also found in other regions of biological significance such as c-myc promoter (Siddiqui-Jain et al., 2002; Seenisamy et al., 2004), hypoxia inducible factor 1 alpha (HIF-alpha) promoter (De Armond et al., 2005), human insulin gene (Catasti et al., 1996) and disease implicated repeats including fragile X syndrome (Fry and Loeb, 1994; Weisman-Shomer et al., 2000; Fojtik et al., 2004). Likewise, mis-regulation of quadruplex associated proteins can lead to severe disorders such as Bloom (Sun et al., 1998) and Werner syndromes (Fry and Loeb, 1999). Moreover, in normal cells, the telomeric tandem repeats are shortened with cell divisions and eventually leads to cell apoptosis in contrast to immortal cancer cells with highly active telomerase that can reverse cell senescence by repeat extension (Carrino et al., 2021). Since G-quadruplex inhibits telomerase activity (Wang et al., 2011; Carrino et al., 2021), understanding the mechanism of quadruplex formation from linearized telomeric DNA induced by small molecule binding is fundamental in anticancer drug design (Neidle, 2010).

**FIGURE 1:**
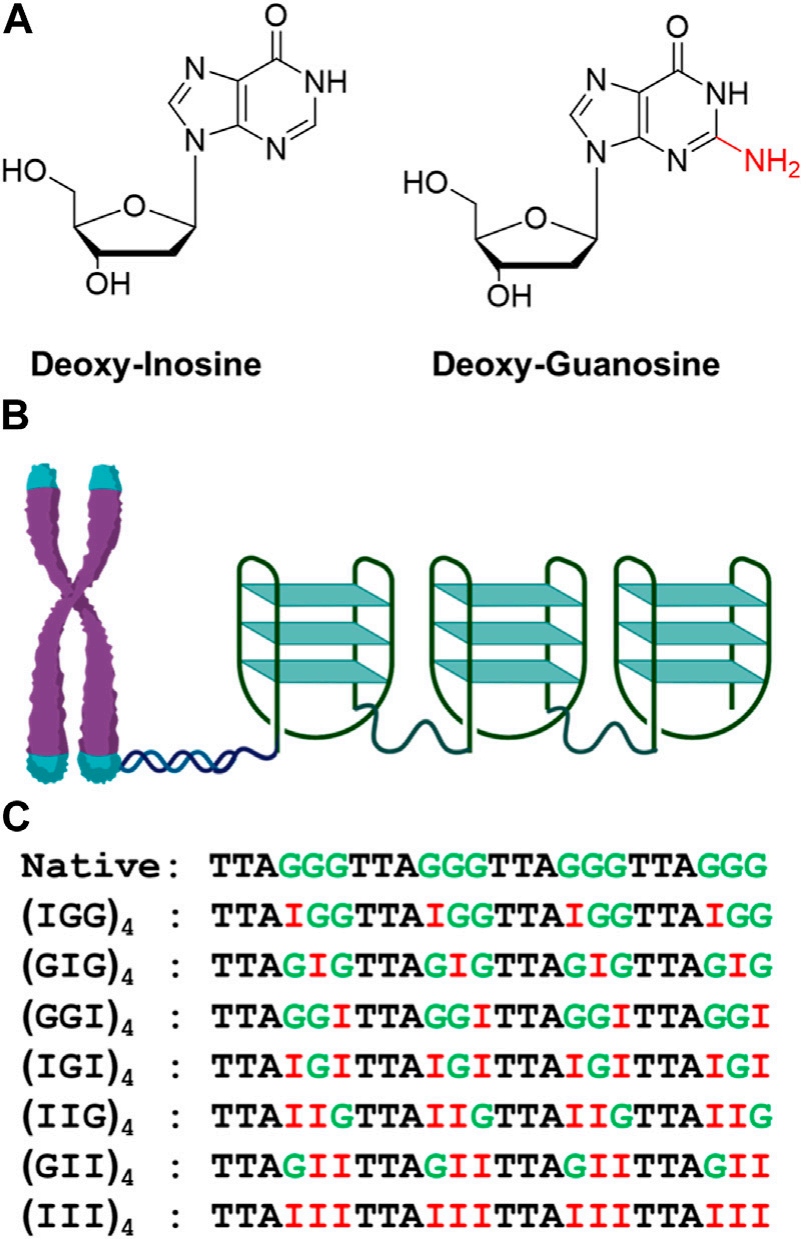
Structures of dI and dG **(A)**. Diagrammatic representation of telomeric repeat sequence forming G-quadruplex consists of antiparallel, parallel, or hybrid topology **(B)** and inosine substituted oligonucleotide used in the folding studies **(C)**.

Deoxyinosine (dI) is a natural DNA nucleotide usually generated from the deamination of deoxyadenosine from the exposure to nitrosative compounds in the environments. The dI residue is read as dG by the replication machinery (Alseth et al., 2014). Due to the lack of N^2^-amino group and the similar electrostatic potentials of the two, the G-to-I mutation was commonly used to study inosine induced Hoogsteen base paring, as well as the effects of ligand binding to both duplex and quadruplex DNAs (Nikolova et al., 2014; Zachary et al., 2019). Inspired by such prospects in cancer intervention, in this work, we performed biophysical and computational studies to explore the conformational features of the telomeric tandem quadruplexes (TTAGGG)_4_ modified with inosines at different positions (Figure 1). The folding landscape of quadruplex formation in the presence of Na^+^ and K^+^ was also investigated and compared through CD, UV-melting and computational modeling studies.

### Materials and methods

The single stranded telomeric DNA and its inosine modified analogs were purchased from IDT (Integrated DNA Technologies). The sequence mimics a small section of the repeat stretch of TTAGGG and its analogs are generated by G_1_, G_2_, G_3_ inosine substitution. DNA samples were reconstituted in nuclease–free water to a final concentration of 1 mM.

### Circular dichroism (CD) spectroscopy studies

CD and CD melting experiments for G4 were performed using the JASCO J-815 CD Spectropolarimeter. Standard CD measurements were carried out under room temperature at 25°C. CD melting utilized temperature gradient spanning from 15°C to 85°C at the rate of 0.5°C/min. A 3 min delay is incorporated for every 5°C increment. Spectra were recorded in a cell path length of 1 mm with an average of 3 scans from 350 nm to 200 nm per interval temperature measurement. Samples were prepared at 10uM DNA concentration in either Na^+^ or K^+^ buffer (same buffer as in UV melting study). Samples were annealed by heating at 95°C then slowly cooled to room temperature over 2 h followed by an overnight incubation at 4°C prior to measurement. The resulting spectra were background subtracted using a buffer blank.

### UV Thermo-melting (T_m_) studies

All thermal stability experiments for G4 were performed on a Cary UV-Vis Multicell Peltier spectrophotometer (Agilent technology). DNA quadruplexes were prepared in either Na^+^ or K^+^ buffer containing 10 mM Na_2_HPO_4_, 10 mM NaH_2_PH_4_, and 100 mM NaCl (for Na^+^ buffer) or 100 mM KCl (for K^+^ buffer) at pH 7, reaching a 600 µL total volume and a final concentration of 1.5 uM. Samples were first annealed at 95°C for 5 min, slowly cooled down to room temperature for 2 h, and incubated at 4°C overnight prior to measurement. Thermal denaturation curve was determined by examining the absorbance as a function of temperature recorded at 290 nm in 1 cm quartz cuvette. Sigmodal fitting with *R* ^2^ of 0.99 was used as a statistical standard to quantify the goodness of the fits (Supplementary Figures S1A, S1B). Each ultraviolet melting curve was measured from 10°C to 85°C at the rate of 0.5°C/min. All measurements were taken four times or with two complete cycles of heating and cooling. Meltwin 3.5 software was then used to obtain the thermodynamic parameters by analyzing the fitted melting curve of DNA quadruplex. The melting temperature of the quadruplex was identified by the maximum in the first derivative of the best fit curves (Supplementary Figures S2A–S2F).

### Computational modeling

3D molecular models were generated for the native and inosine modified sequences to gain structural insights and supplement our experimental observations. Experimentally determined structures of the telomeric repeat sequence in parallel (PDB ID: 1KF1) (Parkinson et al., 2002), antiparallel (PDB ID: 143D) (Wang and Patel, 1993) and hybrid conformations (PDB ID: 5LQG) (Galer et al., 2016) were used as reference structures for the native construct. G-to-I mutations were then performed in MOE (Molecular Operating Environment) [https://www.chemcomp.com/Products.htm] to create 3D structures for each of the inosine modified analogs. The structures were energy minimized to obtain the final structures with inosines accommodation. The structures were then visualized in PyMOL (The PyMOL Molecular Graphics System, Version 2.0 Schrödinger, LLC. https://pymol.org/2/). For hydrogen bond analysis, the donor-acceptor distance cut off and acceptor-donor-hydrogen angle cut off were set at 3.3 Å and 30° respectively.

## Results and discussion

### Circular dichroism spectrum of telomere and its analogs in Na^+^/K^+^ buffer

To understand how the inosine substitutions and the buffer ions affect the conformation of these telomeric repeats, we first investigated the G-quadruplex (GQ) configurations (parallel, antiparallel, and hybrid) using spectroscopic methods. CD has been extensively used to examine the structural confirmation of G-quadruplex with signature absorbance bands occurring between 200 and 300 nm, associated with parallel or antiparallel sugar-phosphate backbones (Gray et al., 2008). Numerous studies reported that the typical antiparallel CD signature demonstrates a positive band at 295 nm and a negative band at 265 nm. In contrast, a parallel CD signature exhibits a 260 nm positive and a 240 nm negative bands (Balagurumoorthy et al., 1992; Guo et al., 1993; Xu et al., 2006).

**FIGURE 2:**
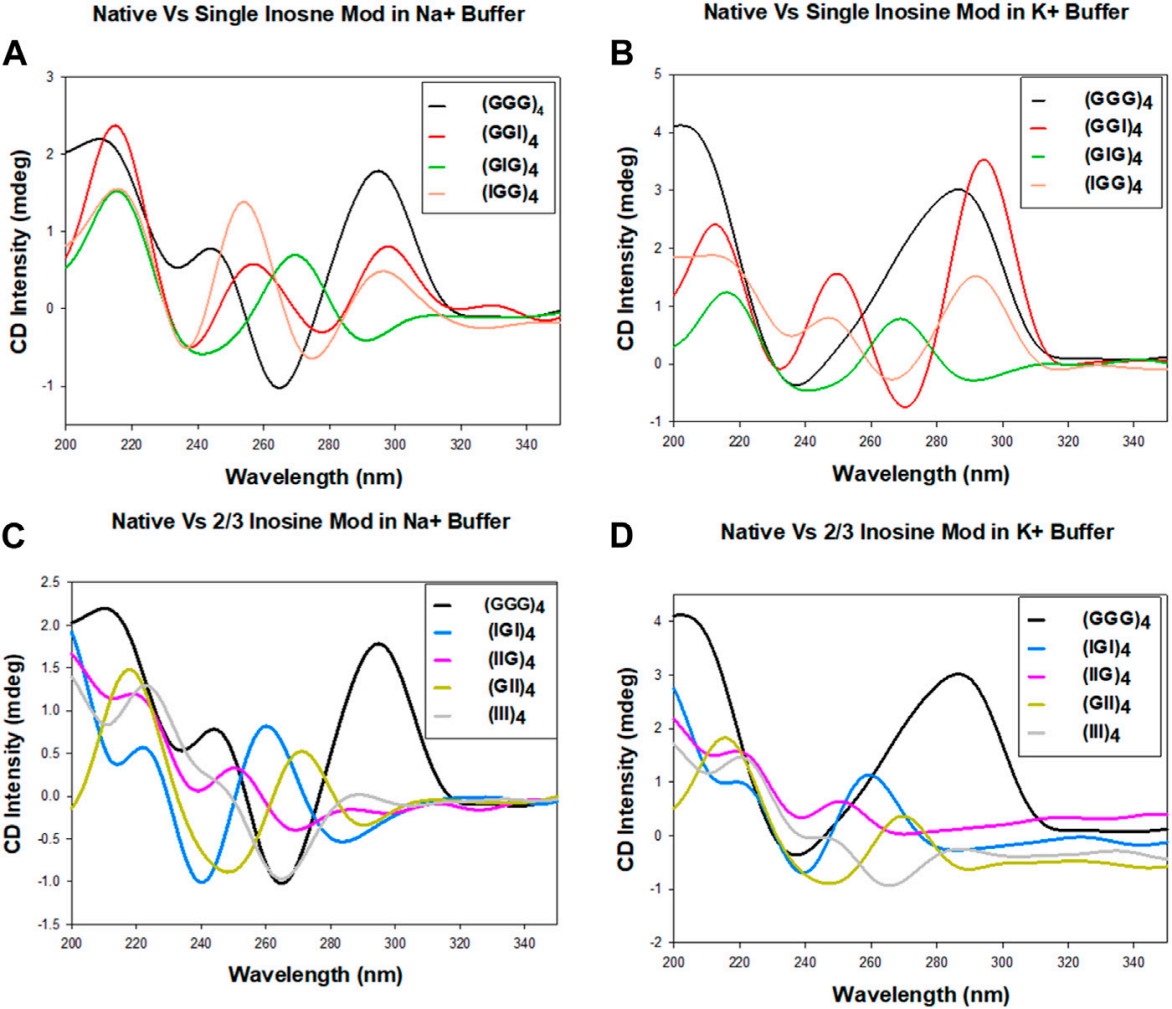
CD spectra of the human telomeric repeat and its inosine substitute analogs in 100 mM Na^+^ or K^+^ containing buffer. Native sequence compared with one, two or three inosine substitutions in Na^+^ **(A,C)** and K^+^ **(B,D)** buffers.

The CD spectra for the unmodified human telomeric repeats and their inosine substituted analogs in the presence of 100 mM Na^+^ or K^+^ monovalent cations are shown in Figure 2. The CD spectra vary significantly from sequence to sequence and also for the different cations. In Na^+^, (GGG)_4_ exhibits bands at +295 nm, −264 nm and +213 nm, consistent with an antiparallel GQ, but in K^+^ it exhibits a hybrid topology with bands at +285 nm and −236 nm (Figures 2A,B). (GGI)_4_ and (IGG)_4_ show very similar behavior in Na^+^ and K^+^ buffers. Though the spectra exhibit several bands for (GGI)_4_: +297 nm, −276 nm, +256 nm, −236 nm, +216 nm, and for (IGG)_4_: +292 nm, −274 nm, +254 nm, −236 nm, +215 nm, the positive band around 295 nm and a slightly shifted negative band at 275 nm is consistent with an antiparallel GQ topology. On the other hand, (GIG)_4_ exhibited bands at +268 nm, −240 nm, +216 nm, suggesting a parallel topology in both cation buffers.

In cases with more than one inosine substitutions, while (IIG)_4_ and (III)_4_ displayed no structural formation, (GII)_4_ exhibited bands at +270 nm, −245 nm, +215 nm, suggesting a parallel topology in both cations. (Figures 2C,D). These results suggest that the formation of antiparallel topology requires at least two consecutive Gs in the repeats. The formation of hybrid topology for native (GGG)_4_ in K^+^ has mixed parallel/antiparallel stranded directionality, consistent with the observation reported by Ambrus and coworkers (Ambrus et al., 2006). Overall, our initial CD data suggest that sequences with consecutive Gs, (GGG)_4_, (IGG)_4_, (GGI)_4_, exhibit antiparallel patterns in CD spectra, while sequences with nonconsecutive Gs, (GIG)_4_, (IGI)_4_, (IIG)_4_, (GII)_4_ (III)_4_, result in parallel or nondetectable topologies.

### UV Thermo-melting (T_m_) studies of inosine G4 strands in Na^+^/K^+^ buffer

**FIGURE 3:**
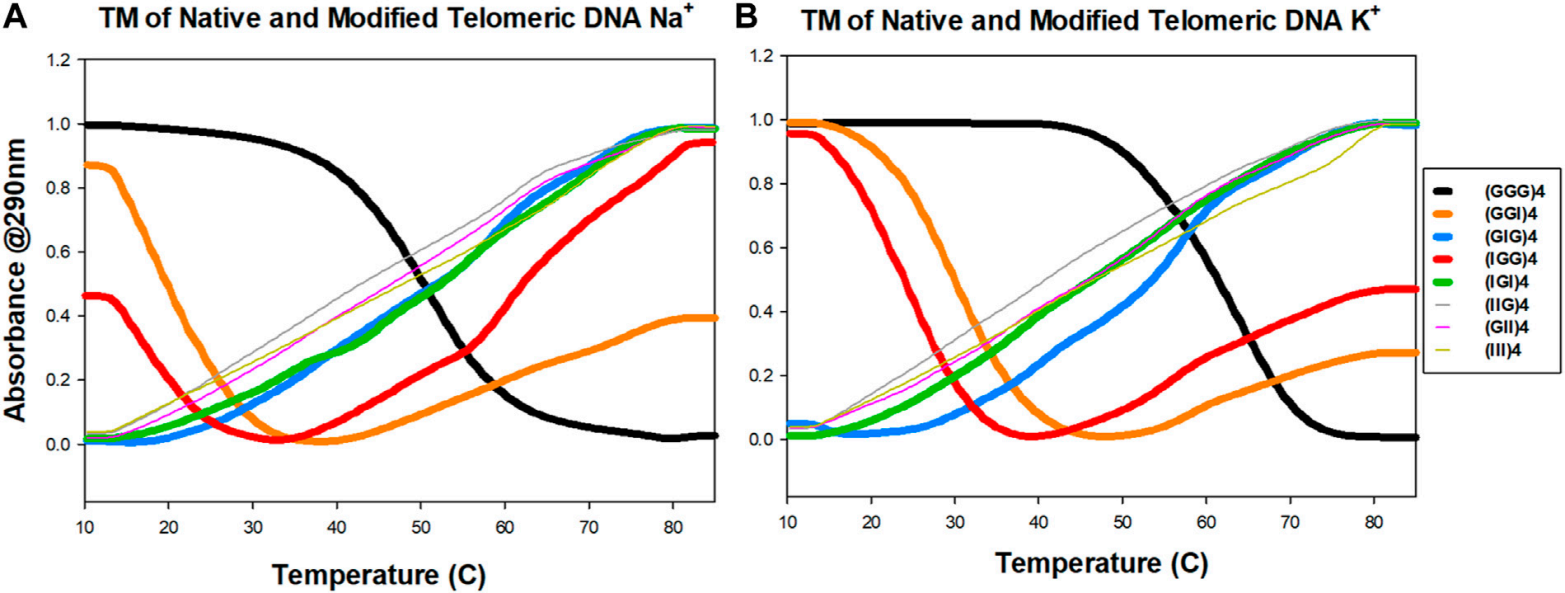
UV melting profiles of telomeric DNA and its analogs. Absorbance at 290 nm as a function of temperature in either 100 mM Na^+^ **(A)** or K^+^ **(B)** ions conditions.

We next investigated the effect of G-to-I substitutions on the stability of potentially formed guanine tetrads by UV-thermal melting analysis in the presence of Na^+^ and K^+^ metal cations. Normalized optical melting curve profiles are shown in Figure 3 for each strand. Among all the examined sequences, native (GGG)_4_ exhibited a sigmodal melting curve with a negative slope in both Na^+^ and K^+^ buffers. K^+^ ions impart more stability as indicated by a higher melting temperature (Figure 3; Table 1). Interestingly, terminal inosine modified DNAs (IGG)_4_ and (GGI)_4_ displayed aberrant sigmodal fitting curves in Na^+^ and K^+^ buffers (Figures 3A,B). In our CD data, these sequences present an antiparallel GQ topology. We speculate that I_1_ and I_3_ substitutions only partially weaken the G-quartets, allowing for a transition stage with a hybrid conformation before it finally melts. Hagen and coworkers reported on 24 nt RNA GQ with inosine substituted on the flanking and showed that a two-step unfolding upon melting and that GI tetrad initiated the unfolding accompanied the loss of one K^+^ ion followed by two-stacked G4 unfolding at higher temperature with the release of the remaining K^+^ ions. Moreover, our results were in agreement with Hagen and coworkers in that inosine induced ∼7°C-8°C decrease in Tm compared to 10°C due to the loss of one H-bond per inosine addition in K^+^ buffer (Hagen et al., 2021). Despite having different sequence in RNAs, the formation of GQ is the same as in DNA.

**TABLE 1:** Results from CD spectroscopy and UV melting experiments for the native telomeric DNA and its inosine modified analogs used in this study.

Sequences	CD-Spe Na^+^	UV-Spe Na^+^	T_m_ (°C) Na^+^	CD-Spe K^+^	UV-Spe K^+^	T_m_ (°C) K^+^
(GGG)_4_	Antiparallel	Sigmodal	50.0	Hybrid	Sigmodal	61.4
(IGG)_4_	Antiparallel	Pseudo-Sigmodal	N/A	Antiparallel	Pseudo-Sigmodal	N/A
(GIG)_4_	Parallel	Sigmodal	52.5	Parallel	Sigmodal	53.5
(GGI)_4_	Antiparallel	Pseudo-Sigmodal	N/A	Antiparallel	Pseudo-Sigmodal	N/A
(IGI)_4_	Parallel	Sigmodal	55.5	Parallel	Sigmodal	46.0
(IIG)_4_	N/A	Non-Sigmodal	N/A	N/A	Non-Sigmodal	N/A
(GII)_4_	Parallel	Non-Sigmodal	N/A	Parallel	Non-Sigmodal	N/A
(III)_4_	N/A	Non-Sigmodal	N/A	N/A	Non-Sigmodal	N/A

(GIG)_4_ showed better stability with a slightly higher melting temperature in K^+^ than in Na^+^ buffer (Table 1). Consistent with the literature, K^+^ ions can stabilize quadruplex structure more effectively than the smaller Na^+^ ions (Largy et al., 2016; Zaccaria et al., 2016). Our CD data shows a parallel configuration for (GIG)_4_, in which the inosines form a weaker quartet and are sandwiched between the two G-tetrads, leading to a stronger quadruplex (refer to the modeling section for details, Figure 5, S6-S8). Study reported by Tanaka and coworkers with 37 nt human telomeric DNA showed that central G-to-I substitution at various positions displayed GQ with different loop arrangements and that the shorter loop is thermodynamically more stable than longer loop. Furthermore, GIG could also be incorporated within a single loop (Atsushi Tanaka and Tetsuro, 2014). Similar scheme with longer (five to seven) human telomeric repeats used in NMR studies by Yue and coworkers showed that selectively modifying the central guanosine with inosine resulted in (3 + 1) form 2 GQ with one extended double chain-reversal or propeller and two edgewise loops. The group further demonstrated that the propeller loop could harbor one or more GGGTTA repeats. This is consistent with our results that GIG contained stable parallel TTA propeller in four repeats human telomeric. Moreover, Tm value detected for our native (GGG)_4_ in K^+^ is 61.4°C that is comparable to the reported Tm of 62.0°C by Yue’s group (Yue et al., 2011).

Sequences with more than one inosine modification exhibited non-sigmodal behavior except (IGI)_4_, which shows a positive sigmodal melting curve in both buffer ions. This data further supported our assumption that the formation and the stabilization of quadruplex are modification site and ion dependent. In other words, positional lesion in the telomeric DNA can have varying extent of tetrad disruption. Overall, our current UV melting data suggests that inosine substitution potentially distorts the folding and unfolding landscape of the telomeric quadruplex and the instability of the conformation is correlated to the number of inosine substitutions, with the weakest being (III)_4_.

### CD melting data for the telomere and its analogs in Na^+^/K^+^ buffer

**FIGURE 4:**
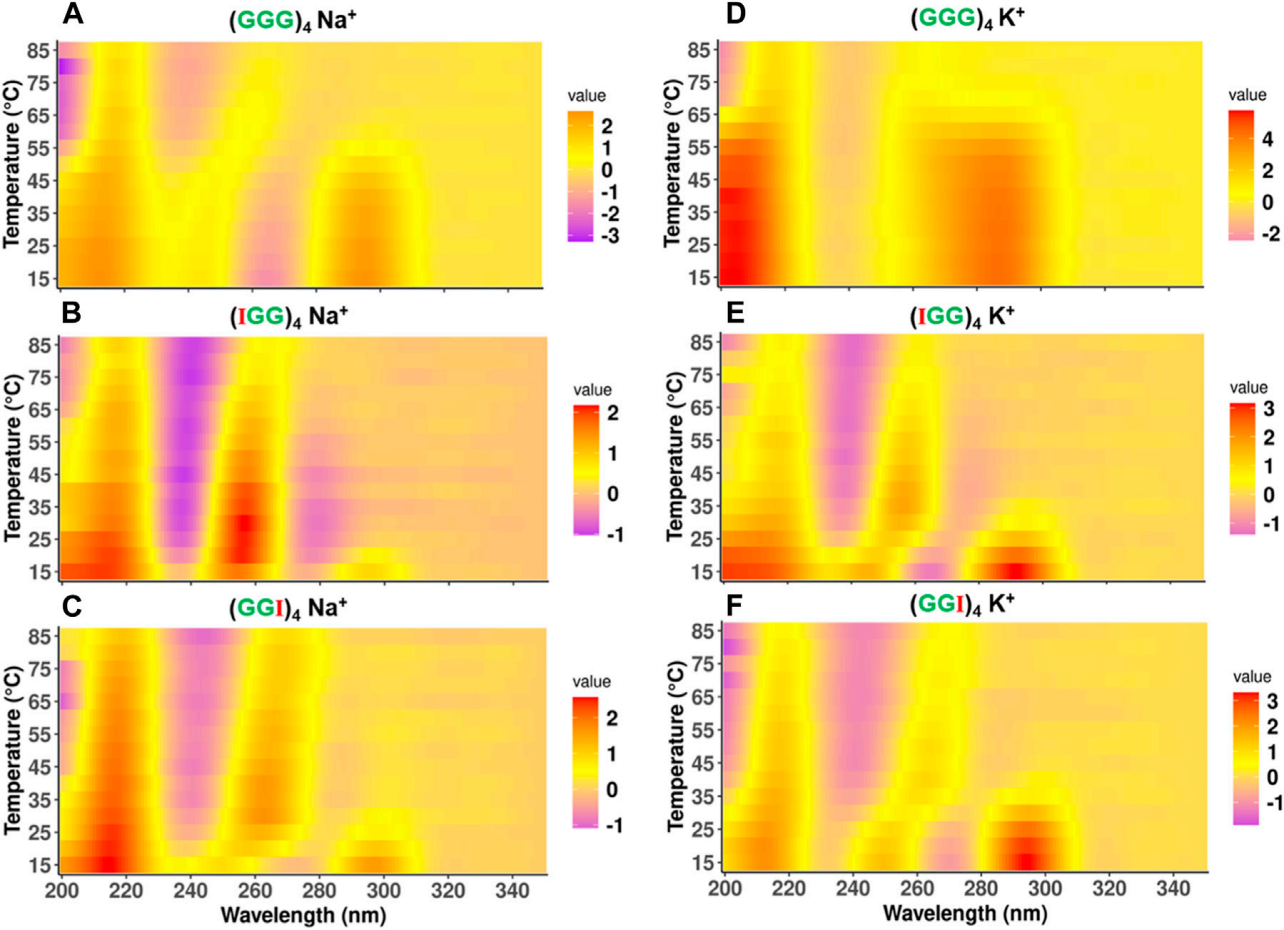
Comparison of CD melting spectra of telomeric DNA and its terminal modified analogs (GGG)_4_, (IGG)_4_, (GGI)_4_. Topological configuration of DNAs reported at the wavelength of 200nm–300 nm with mean residue ellipticity as function of temperature in the presence of 100 mM Na^+^ **(A–C)** and 100 mM K^+^ **(D–F)**. The intensity of ellipticity has unit of degree × cm^2^ × dmol^−1^.

While UV T_m_ data informs stability via melting temperature assessment, it gives little information regarding the stability of topological conformations during the melting process. To further understand the conformational stability associated with unfolding of these telomeric repeat sequences, we obtained the CD melting spectra for a temperature range 15°C–85°C. The spectral profiles as a function of temperature are shown as a heatmap in Figure 4, where the color represents the standardized CD spectra intensity, a purple band indicates a negative peak and a red band indicates a positive peak. This representation provides a conspicuous view to observe transitions in topologies. The classical representation of CD spectra profiles is shown in Supplementary Figure S5A for Na^+^ and Supplementary Figure S5B for K^+^ for reference.

For the native (GGG)_4_ in 100 mM Na^+^, the spectra show a transition from antiparallel to parallel GQ topology, as the (+295nm, −264 nm) shifts to a (+262 nm, −240 nm) band. This transition happens between 50°C and 60°C range, which is consistent with the observed melting temperature in our UV melting data (Table 1). On the other hand, in 100 mM K^+^, (GGG)_4_ delineated two stages of transformation, characterized by a hybrid conformation over temperature range from 15°C to 60°C and antiparallel over 65°C–70°C with a transitioning temperature around 60°C as well, consistent with our UV melting (Table 1). This thermal melting is higher than (GGG)_4_ in 100 mM Na^+^. It eventually folds into a parallel topology between 75°C–85°C (Figure 4D).

Interestingly, for (IGG)_4_ and (GGI)_4_ in Na^+^, the antiparallel state is less prominent and the transition from antiparallel to parallel GQ topology happens at a much lower temperature than the native one, between 20°C–25°C for (IGG)_4_ (Figure 4B), and 30°C–35°C for (GGI)_4_ (Figure 4C). The parallel conformation is still strong even at higher temperatures, as indicated by the (+260 nm, −245 nm) bands. In K^+^, while both (IGG)_4_ and (GGI)_4_ follow the same trend as in Na^+^ buffer, the transition temperature is higher, between 35°C–40°C for (IGG)_4_ (Figure 4E), and between 45°C–50°C for (GGI)_4_ (Figure 4F). (GIG)_4_ tends to maintain a stable parallel topology over the entire temperature range of 15°C–85°C (Supplementary Figures S3A, S3D) in both Na^+^ and K^+^ buffers. For cases with multiple inosine substitutions, (IGI)_4_, and (GII)_4_ showed stable parallel topology over the temperature range of 15°C–85°C (Supplementary Figures S3B, S3C and S3E, S3F), while (IIG)_4_ and (III)_4_ showed no clear conformation (Supplementary Figures S4A–D), which is consistent with UV melting data where no clear structural transitions were observed for both Na^+^ and K^+^ buffers.

### *In silico* modeling studies of inosine substituted G-quaduplexes

**FIGURE 5:**
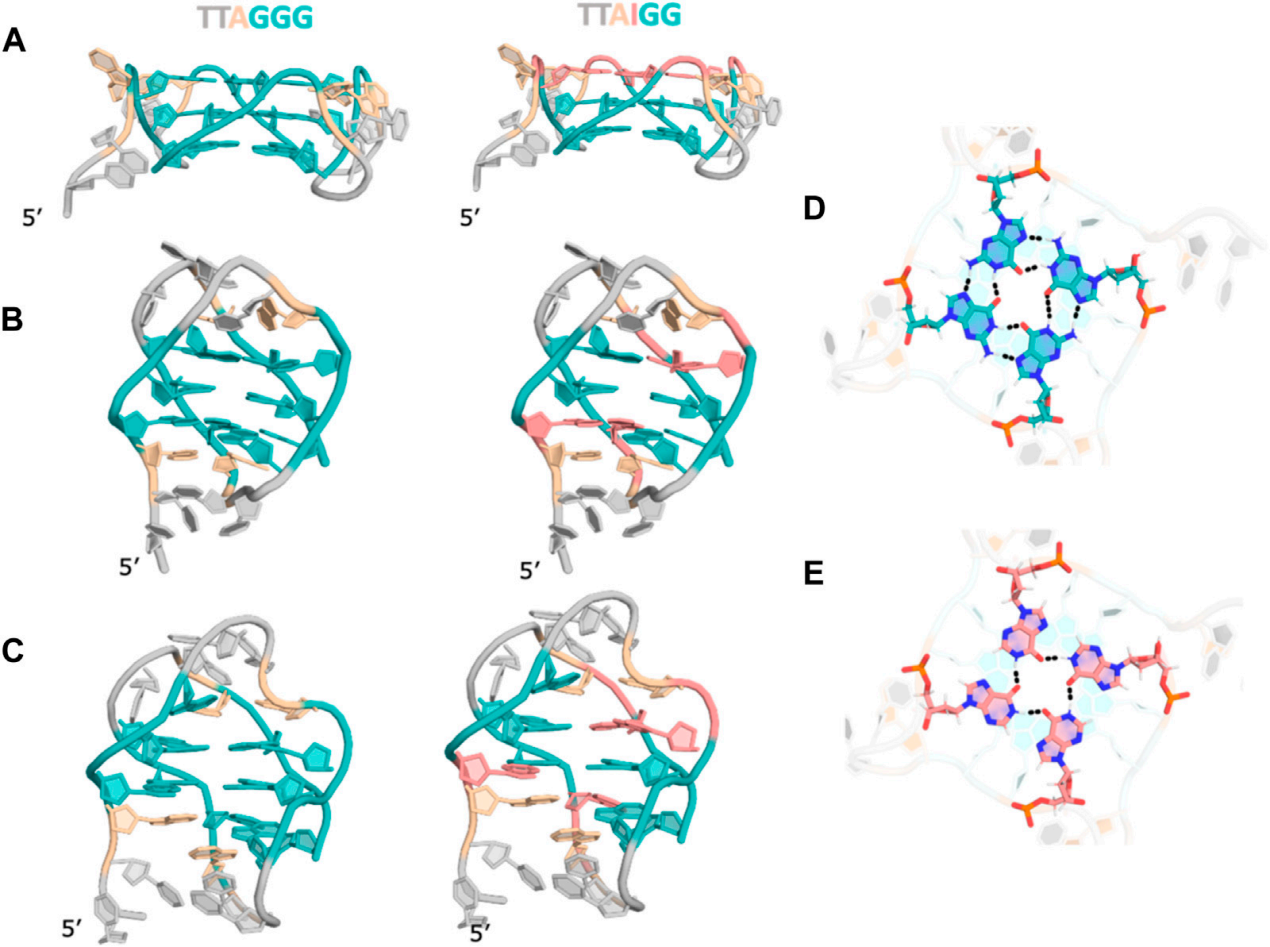
Structural models for (GGG)_4_ and (IGG)_4_ in **(A)** parallel **(B)**. Antiparallel **(C)**. Hybrid topologies. **(D)**. Cross-sectional view of a G-quartet showing h-bonding pattern. **(E)**. Cross-sectional view of an “I-quartet” showing hydrogen bonding patterns. For clarity, ions are not shown.

In light of the various experimental results, we employed *in silico* modeling studies to explore the structures that these sequences could adopt. Specifically, we used experimentally determined structures of telomeric repeats as reference and generated inosine substituted structures for parallel, antiparallel and hybrid topologies (Supplementary Figures S6–S8). The hybrid conformation is a G-quartet dimer from previous NMR studies in the presence of K^+^ ions at neutral pH (Brahmachari, 1994). In the parallel form, the substituted inosines in the GGG repeat sequence, at any of the three positions, end up as part of the same G-quartet. However, in the antiparallel topology, G-I mutations at G_1_ and G_3_ are distributed over two quartets while a mutation at G_2_ leads to an “I-quartet”. In the hybrid topology with the two quartet conformation, inosine substitutions also result in the inosines either distributed between the quartets or as part of the connecting loops or stabilizing triplets in the ends (Figures 5A–C). Within the G-quartets of the quadruplex, a G-I mutation leads to a loss of one hydrogen bond (as shown in Figures 5D,E).

In our experiments we observed that the native quadruplex forms an antiparallel topology and a hybrid topology in the presence of Na^+^ and K^+^ ions respectively, in agreement with the previously determined structures (Brahmachari, 1994; Zhang et al., 2003; Rujan et al., 2005; Antonacci et al., 2007; Tucker et al., 2018). Interestingly, the CD melting data shows that (IGG)_4_, (GGI)_4_ transition from antiparallel to parallel quadruplex at higher temperatures in both cation buffers. The structure models of these sequences suggest that the one I-quartet in parallel quadruplexes is better accommodated compared to mixed IG-quartets in anti-parallel structures. Extending this same rationale, while (GIG)_4_ starts off and continues to maintain a parallel topology at higher temperatures, our modeling study suggests that it has the potential to be stabilized as an antiparallel quadruplex as well. Both (GII)_4_, (IGI)_4_ also maintain their parallel topologies at higher temperatures, showing that multiple I-quartets are well tolerated in the structure as long as there is one intact G-quartet, while (III)_4_ does not show any CD spectra, consistent with quadruplex formation, suggesting all I-quartets cannot hold the structure in either parallel or antiparallel topologies. An exception is the (IIG)_4_ strand which behaves like (III)_4_ instead of (GII)_4_ even though (GII)_4_ and (IIG)_4_ both are expected to form symmetric structures. The loop nucleotides adjacent (IIG)_4_ are adenosines (As) while those adjacent the I-quartet in (GII)_4_ are thymines (Ts). This difference could be a contributing factor towards formation or melting of these structures.

## Conclusion

Overall, our study suggests that inosine substitutions in the telomere tandem repeats of (TTAGGG)n sequence could alter local base pairings within the core of the quadruplex and affect their overall conformation and stability in a site-dependent fashion with different ions, specifically contingent on the number of Gs per quartet. We demonstrated that by only replacing G_1_ or G_3_ terminal guanine in the repeats, i.e., (IGG)_4_ or (GGI)_4_, the quadruplex formation is well retained, although the structure is less stable and goes through a potential conformational change during melting. The replacement of central guanine G_2_ with inosine does not contribute to quadruplex formation (GIG)_4_. Two or three inosines disfavor quadruplex formation. Replacement with central inosine and two or more inosines in the repeats (GII)_4_, (IGI)_4_ (IIG)_4_ and (III)_4_ do not show any specific quadruplex fingerprints in our CD data. A simple model is that terminal guanine lesions can be compensated by the other two G-quartets and stabilized by Na^+^ or K^+^ ions, while this is not the case with inosine substitution in the central guanine or more than one guanine.

Furthermore, it has been previously reported that both extracellular and intracellular Na^+^ and K^+^ cations can facilitate hydrophobic stacking of two quartets by reducing the electronic repulsion induced by central oxygen atoms. Therefore, the polarity and the relative orientations of stacking quartets can modulate spectra outcomes (Debmalya et al., 2016). The biological significance of these structures is linked to the stability and extent of interaction with the telomerase (Blackburn et al., 1997). Based on our UV-melting results specifically for native and central inosine modified sequences, the structures formed in K^+^ generally have higher stability than the ones in Na^+^, which is consistent with previous literature (Largy et al., 2016; Zaccaria et al., 2016). However, this is not the case when two terminal Gs are mutated to I, which might be critical when considering that the stability of G-quadruplex can also influence their interactions with telomerase. Less stable quadruplexes allow telomerase to reform Watson-Crick pairing with RNA template, leading to further destabilization (Wang et al., 2011; Carrino et al., 2021). With an expanded base pairing preference (inosines can base pair with T, C and A), inosine can contribute significantly to this structural destabilization and consequently duplex formation. In summary, our current study demonstrates that G-quadruplexes can be reorganized, destabilized or structurally transformed by inosine substitutions, all of which could affect the affinity to bind with small molecule drug targeting G4.

## Data Availability

The original contributions presented in the study are included in the article/Supplementary Material, further inquiries can be directed to the corresponding authors.

## References

[B1] Alseth I. Dalhus B. Bjoras M. (2014). Inosine in DNA and RNA. Curr. Opin. Genet. Dev. 26, 116–123. 10.1016/j.gde.2014.07.008 25173738

[B2] Ambrus A. Chen D. Dai J. Bialis T. Jones R. A. Yang D. (2006). Human telomeric sequence forms a hybrid-type intramolecular G-quadruplex structure with mixed parallel/antiparallel strands in potassium solution. Nucleic Acids Res. 34, 2723–2735. 10.1093/nar/gkl348 16714449 PMC1464114

[B3] Antonacci C. Chaires J. B. Sheardy R. D. (2007). Biophysical characterization of the human telomeric (TTAGGG)4 repeat in a potassium solution. Biochemistry 46, 4654–4660. 10.1021/bi602511p 17381076

[B4] Atsushi Tanaka J. C. Tetsuro MAJIMA Majima T. (2014). Folding and structural polymorphism of gquadruplex formed from a long telomeric sequence containing six GGG tracts. R. Soc. Chem. Adv. 4, 59071–59077. 10.1039/c4ra08053j

[B5] Balagurumoorthy P. Brahmachari S. K. Mohanty D. Bansal M. Sasisekharan V. (1992). Hairpin and parallel quartet structures for telomeric sequences. Nucleic Acids Res. 20, 4061–4067. 10.1093/nar/20.15.4061 1508691 PMC334088

[B6] Blackburn E. Bhattacharyya A. Gilley D. Kirk K. Krauskopf A. Mceachern M. (1997). The telomere and telomerase: how do they interact? Ciba Found. Symp. 211, 2–13. 10.1002/9780470515433.ch2 9524748

[B7] Bodnar A. G. Ouellette M. Frolkis M. Holt S. E. Chiu C. P. Morin G. B. (1998). Extension of life-span by introduction of telomerase into normal human cells. Science 279, 349–352. 10.1126/science.279.5349.349 9454332

[B8] Brahmachari P. B. A. S. K. Brahmachari S. (1994). Structure and stability of human telomeric sequence. J. Biol. Chem. 269, 21858–21869. 10.1016/s0021-9258(17)31882-3 8063830

[B9] Bryan T. M. (2020). G-quadruplexes at telomeres: friend or foe? Molecules 25, 3686. 10.3390/molecules25163686 32823549 PMC7464828

[B10] Carrino S. Hennecker C. D. Murrieta A. C. Mittermaier A. (2021). Frustrated folding of guanine quadruplexes in telomeric DNA. Nucleic Acids Res. 49, 3063–3076. 10.1093/nar/gkab140 33693924 PMC8034632

[B11] Catasti P. Chen X. Moyzis R. K. Bradbury E. M. Gupta G. (1996). Structure-function correlations of the insulin-linked polymorphic region. J. Mol. Biol. 264, 534–545. 10.1006/jmbi.1996.0659 8969303

[B12] De Armond R. Wood S. Sun D. Hurley L. H. Ebbinghaus S. W. (2005). Evidence for the presence of a guanine quadruplex forming region within a polypurine tract of the hypoxia inducible factor 1α promoter. Biochemistry 44, 16341–16350. 10.1021/bi051618u 16331995

[B13] Debmalya B. Arachchilage G. M. Soumitra B. (2016). Metal cations in G-quadruplex folding and stability. Front. Chem. 4, 38. 10.3389/fchem.2016.00038 27668212 PMC5016522

[B14] Fojtik P. Kejnovska I. Vorlickova M. (2004). The guanine-rich fragile X chromosome repeats are reluctant to form tetraplexes. Nucleic Acids Res. 32, 298–306. 10.1093/nar/gkh179 14718550 PMC373289

[B15] Fry M. Loeb L. A. (1994). The fragile X syndrome d(CGG)n nucleotide repeats form a stable tetrahelical structure. Proc. Natl. Acad. Sci. U. S. A. 91, 4950–4954. 10.1073/pnas.91.11.4950 8197163 PMC43907

[B16] Fry M. Loeb L. A. (1999). Human werner syndrome DNA helicase unwinds tetrahelical structures of the fragile X syndrome repeat sequence d(CGG). J. Biol. Chem. 274, 12797–12802. 10.1074/jbc.274.18.12797 10212265

[B17] Galer P. Wang B. Sket P. Plavec J. (2016). Reversible pH switch of two-quartet G-quadruplexes formed by human telomere. Angew. Chem. Int. Ed. Engl. 55, 1993–1997. 10.1002/anie.201507569 26836334

[B18] Gray D. M. Wen J. D. Gray C. W. Repges R. Repges C. Raabe G. (2008). Measured and calculated CD spectra of G-quartets stacked with the same or opposite polarities. Chirality 20, 431–440. 10.1002/chir.20455 17853398

[B19] Guo Q. Lu M. Kallenbach N. R. (1993). Effect of thymine tract length on the structure and stability of model telomeric sequences. Biochemistry 32, 3596–3603. 10.1021/bi00065a010 8466901

[B20] Hagen T. Laski A. Brummer A. Pruska A. Schlosser V. Clery A. (2021). Inosine substitutions in RNA activate latent G-quadruplexes. J. Am. Chem. Soc. 143, 15120–15130. 10.1021/jacs.1c05214 34520206

[B21] Hahn W. C. Stewart S. A. Brooks M. W. York S. G. Eaton E. Kurachi A. (1999). Inhibition of telomerase limits the growth of human cancer cells. Nat. Med. 5, 1164–1170. 10.1038/13495 10502820

[B22] Harley C. B. Futcher A. B. Greider C. W. (1990). Telomeres shorten during ageing of human fibroblasts. Nature 345, 458–460. 10.1038/345458a0 2342578

[B23] Largy E. Mergny J. L. Gabelica V. (2016). Role of alkali metal ions in G-quadruplex nucleic acid structure and stability. Met. Ions Life Sci. 16, 203–258. 10.1007/978-3-319-21756-7_7 26860303

[B24] Liyanage V. R. Jarmasz J. S. Murugeshan N. Del Bigio M. R. Rastegar M. Davie J. R. (2014). DNA modifications: function and applications in normal and disease States. Biol. (Basel) 3, 670–723. 10.3390/biology3040670 PMC4280507 25340699

[B25] Mergny J. L. Riou J. F. Mailliet P. Teulade-Fichou M. P. Gilson E. (2002). Natural and pharmacological regulation of telomerase. Nucleic Acids Res. 30, 839–865. 10.1093/nar/30.4.839 11842096 PMC100331

[B26] Neidle S. (2010). Human telomeric G-quadruplex: the current status of telomeric G-quadruplexes as therapeutic targets in human cancer. FEBS J. 277, 1118–1125. 10.1111/j.1742-4658.2009.07463.x 19951354

[B27] Nikolova E. N. Stull F. Al-Hashimi H. M. (2014). Guanine to inosine substitution leads to large increases in the population of a transient G·C hoogsteen base pair. Biochemistry 53, 7145–7147. 10.1021/bi5011909 25339065 PMC4245982

[B28] Parkinson G. N. Lee M. P. Neidle S. (2002). Crystal structure of parallel quadruplexes from human telomeric DNA. Nature 417, 876–880. 10.1038/nature755 12050675

[B29] Rujan I. N. Meleney J. C. Bolton P. H. (2005). Vertebrate telomere repeat DNAs favor external loop propeller quadruplex structures in the presence of high concentrations of potassium. Nucleic Acids Res. 33, 2022–2031. 10.1093/nar/gki345 15817566 PMC1074753

[B30] Seenisamy J. Rezler E. M. Powell T. J. Tye D. Gokhale V. Joshi C. S. (2004). The dynamic character of the G-quadruplex element in the c-MYC promoter and modification by TMPyP4. J. Am. Chem. Soc. 126, 8702–8709. 10.1021/ja040022b 15250722

[B31] Siddiqui-Jain A. Grand C. L. Bearss D. J. Hurley L. H. (2002). Direct evidence for a G-quadruplex in a promoter region and its targeting with a small molecule to repress c-MYC transcription. Proc. Natl. Acad. Sci. U. S. A. 99, 11593–11598. 10.1073/pnas.182256799 12195017 PMC129314

[B32] Sun H. Karow J. K. Hickson I. D. Maizels N. (1998). The Bloom's syndrome helicase unwinds G4 DNA. J. Biol. Chem. 273, 27587–27592. 10.1074/jbc.273.42.27587 9765292

[B33] Travers A. Muskhelishvili G. (2015). DNA structure and function. FEBS J. 282, 2279–2295. 10.1111/febs.13307 25903461

[B34] Tucker B. A. Hudson J. S. Ding L. Lewis E. Sheardy R. D. Kharlampieva E. (2018). Stability of the Na(+) form of the human telomeric G-quadruplex: role of adenines in stabilizing G-quadruplex structure. ACS Omega 3, 844–855. 10.1021/acsomega.7b01649 30023791 PMC6045420

[B35] Wang Q. Liu J. Q. Chen Z. Zheng K. W. Chen C. Y. Hao Y. H. (2011). G-quadruplex formation at the 3' end of telomere DNA inhibits its extension by telomerase, polymerase and unwinding by helicase. Nucleic Acids Res. 39, 6229–6237. 10.1093/nar/gkr164 21441540 PMC3152327

[B36] Wang Y. Patel D. J. (1993). Solution structure of the human telomeric repeat d[AG3(T2AG3)3] G-tetraplex. Structure 1, 263–282. 10.1016/0969-2126(93)90015-9 8081740

[B37] Weisman-Shomer P. Naot Y. Fry M. (2000). Tetrahelical forms of the fragile X syndrome expanded sequence d(CGG) are destabilized by two heterogeneous nuclear ribonucleoprotein-related telomeric DNA-binding proteins. J. Biol. Chem. 275, 2231–2238. 10.1074/jbc.275.3.2231 10636930

[B38] Wellinger R. J. Sen D. (1997). The DNA structures at the ends of eukaryotic chromosomes. Eur. J. Cancer 33, 735–749. 10.1016/s0959-8049(97)00067-1 9282112

[B39] Xu Y. Noguchi Y. Sugiyama H. (2006). The new models of the human telomere d[AGGG(TTAGGG)3] in K+ solution. Bioorg Med. Chem. 14, 5584–5591. 10.1016/j.bmc.2006.04.033 16682210

[B40] Yue D. J. Lim K. W. Phan A. T. (2011). Formation of (3+1) G-quadruplexes with a long loop by human telomeric DNA spanning five or more repeats. J. Am. Chem. Soc. 133, 11462–11465. 10.1021/ja204197d 21702440

[B41] Zaccaria F. Paragi G. Fonseca Guerra C. (2016). The role of alkali metal cations in the stabilization of guanine quadruplexes: why K(+) is the best. Phys. Chem. Chem. Phys. 18, 20895–20904. 10.1039/c6cp01030j 27185388

[B42] Zachary E. Ferris Q. L. Markus W. Germann (2019). Substituting inosine for guanosine in DNA: structural and dynamic consequences. Nat. Product. Commun. 14, 10.1177/1934578x19850032

[B43] Zhang X. Y. Cao E. H. Zhang Y. Chou C. Bai C. (2003). K+ and Na+-induced self-assembly of telomeric oligonucleotide d(TTAGGG)n. J. Biomol. Struct. Dyn. 20, 693–701. 10.1080/07391102.2003.10506886 12643772

