# Supplementary material for: Structural effects of inosine substitution in telomeric DNA quadruplex
Source: Front Chem. 2024 Jan 19;12:1330378. doi: 10.3389/fchem.2024.1330378 (PMC10834636; doi:10.3389/fchem.2024.1330378)
Supplement: Supplementary file 1 [file DataSheet1.docx]

**Supporting Information**

**Structural Effects of Inosine Substitution in Telomeric DNA Quadruplex**

Ya Ying Zheng,^a,b^ Ricky Dartawan,^a,b^ Yuhan Wu,^a,b^ Chengze Wu,^a,b^ Hope Zhang,^a,b^ Jeanne Lu,^a,b^ Ashley Hu,^a,b^ Sweta Vangaveti,^b,*^ and Jia Sheng^a,b,^*

^a^Department of Chemistry, and ^b^The RNA Institute, University at Albany, State University of New York, 1400 Washington Avenue, Albany, NY 12222, USA

*Corresponding author: jsheng@albany.edu (J.S.), svangaveti@albany.edu (S.V.)

**
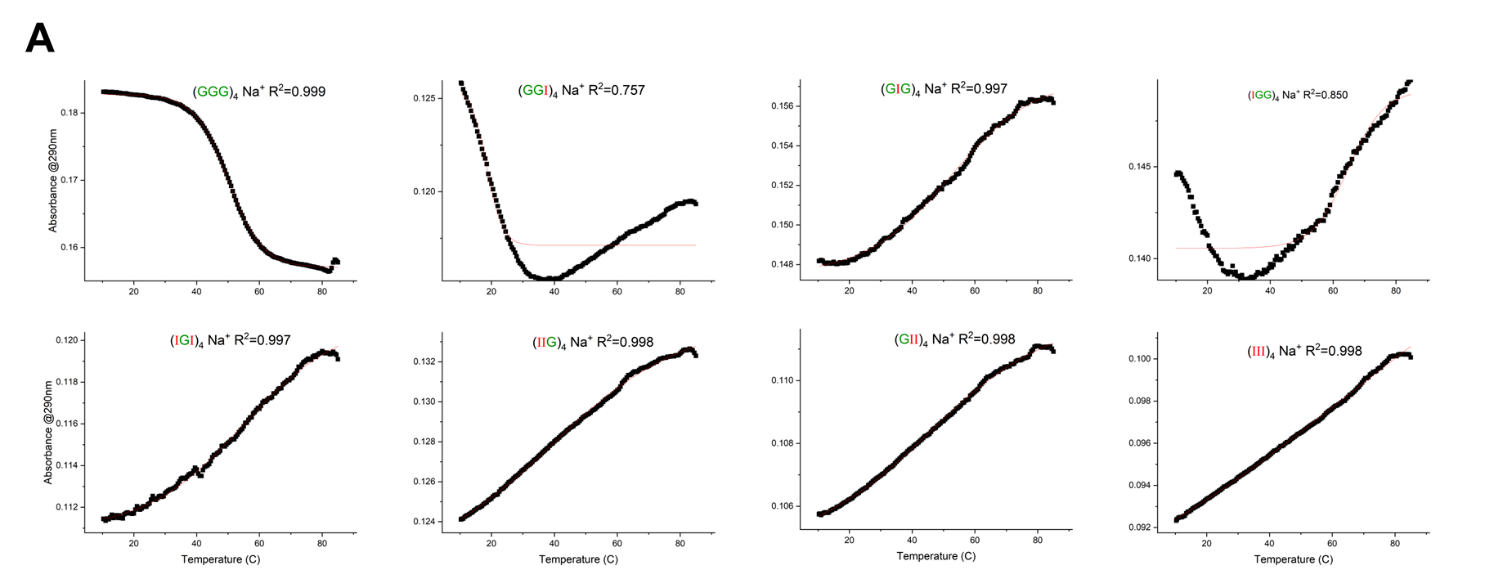
**


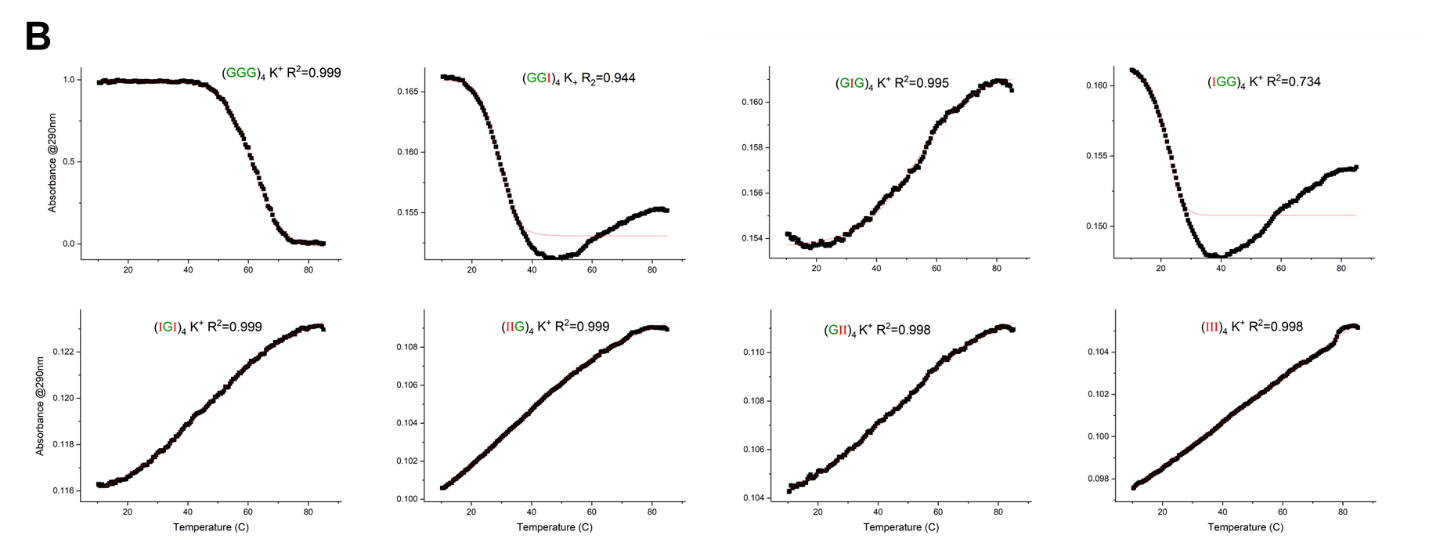


**S1: Results of UV-Melting profiles for each DNA studied by sigmodal fitting**

Among all DNAs tested, the most ideal sigmodal fittings were observed for (GGG)_4_, (GIG)_4_ and (IGI)**_4_** in 100mM Na^+^ and K^+^ buffers **(A, B).**


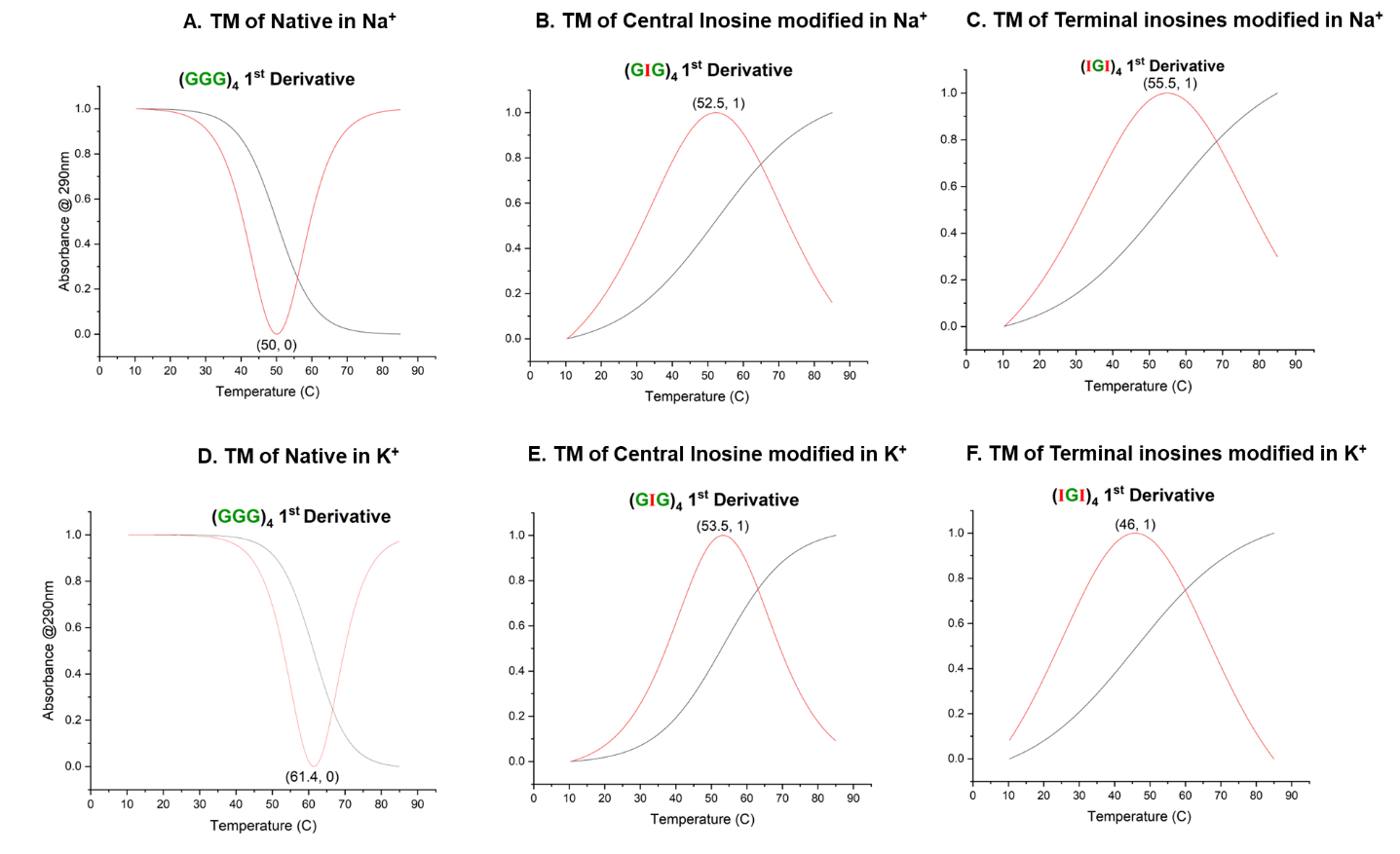


**S2: Analyzed Melting temperature from the best sigmodal fitting curves**

The maximum in the first derivative of absorbance with respect to temperature in the ultraviolet at 290nm is the melting temperature for the detectable configuration, quadruplex or duplex. Top panel showed the most ideal UV-melting profiles in physiological salts100mM Na^+^, bottom panel showed in 100mM K^+^.


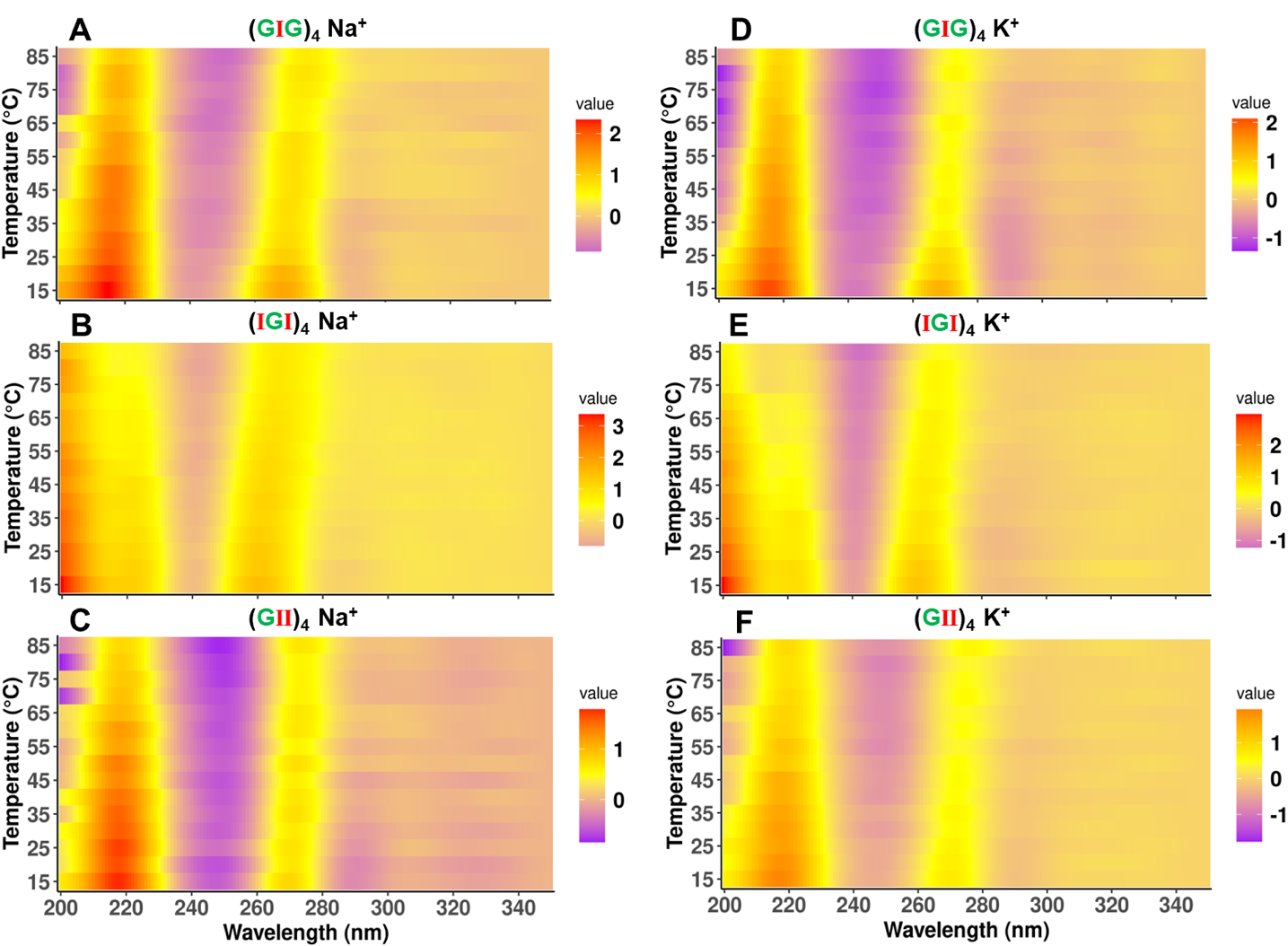


**S3:** The CD melting spectra of (GIG)_4_, (IGI)_4_, (GII)_4_ in 100mM Na^+^ are shown in the left panels, and those in 100mM K^+^ are shown in the right panels, with temperatures ranging from 15ºC to 85ºC. Stable parallel topologies were observed for these three analogs in all temperature gradients.


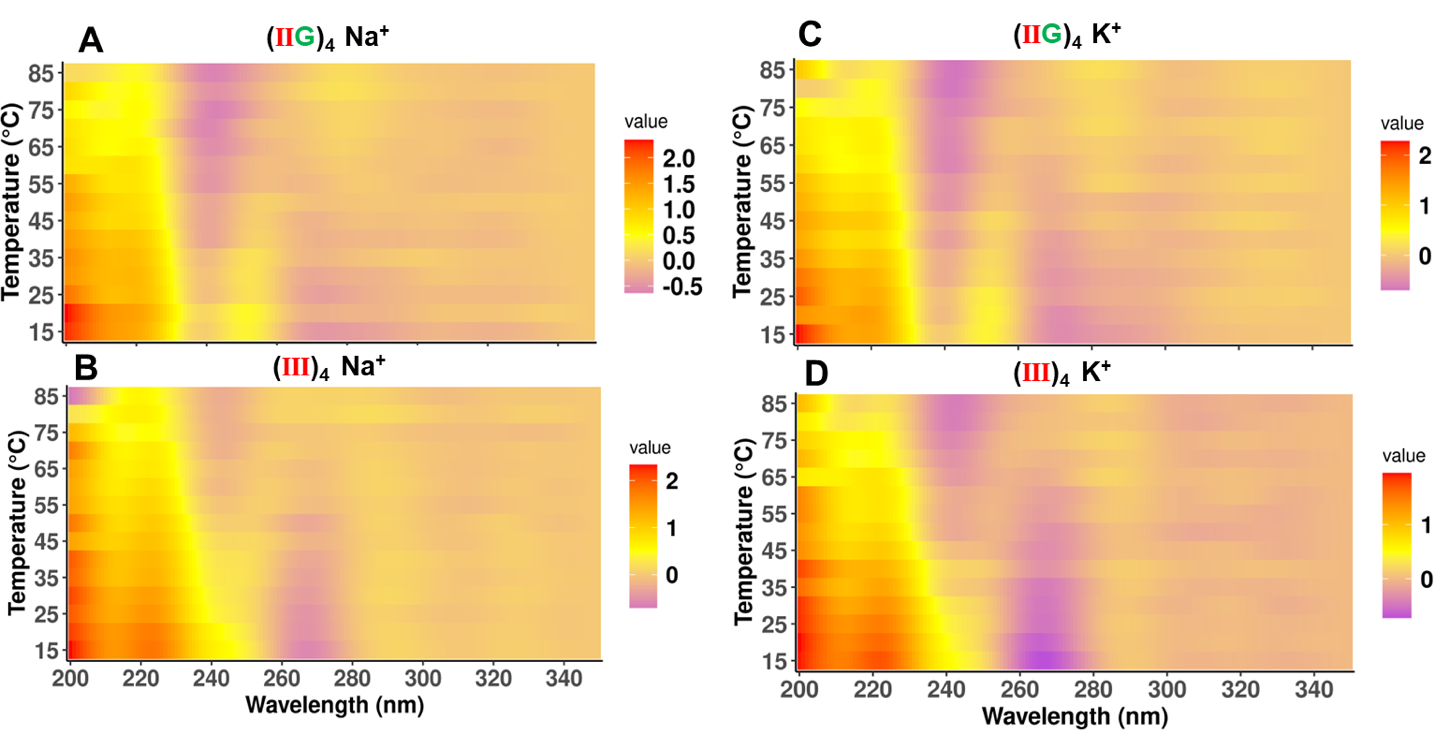


**S4:** The CD melting spectra of (IIG)_4_ and (III)_4_ in 100mM Na^+^ are shown in the left panels, and those in 100mM K^+^ are shown in the right panels, with temperatures ranging from 15ºC to 85ºC. No obvious structures were detected for these two analogs at all temperature gradients.


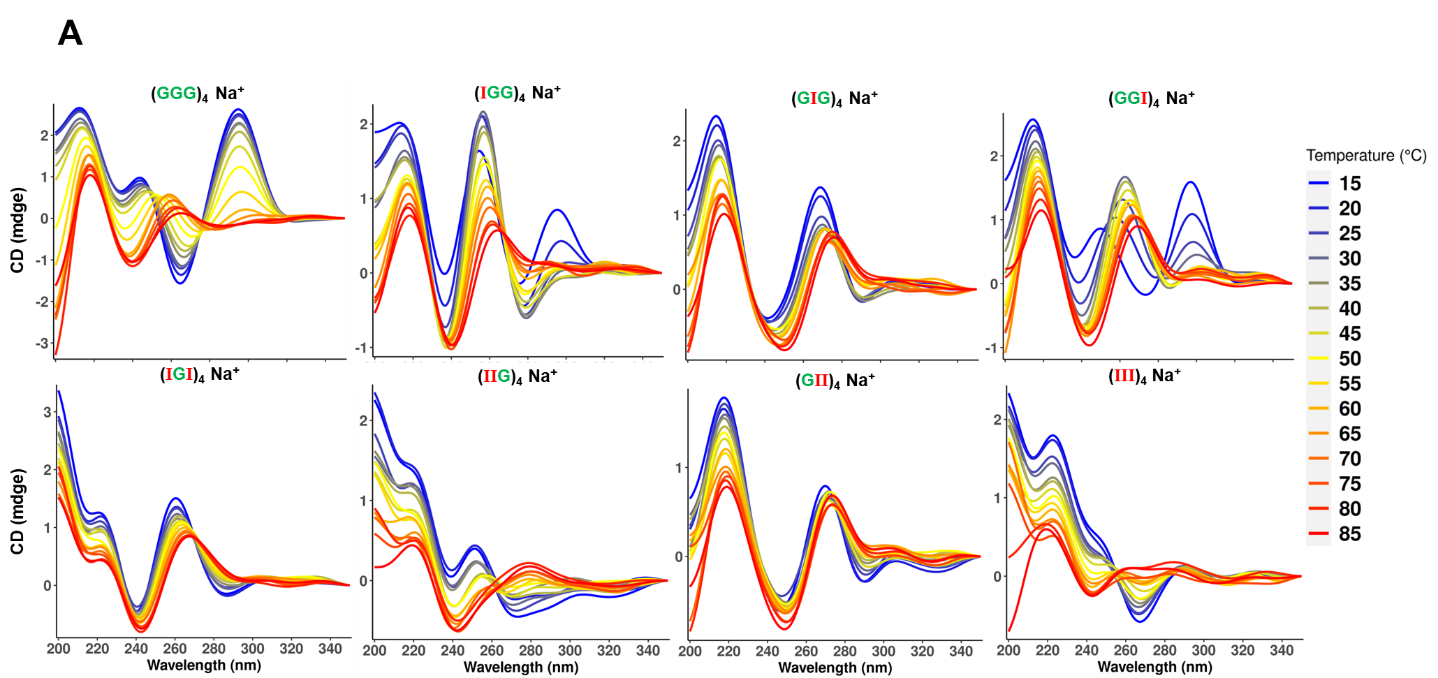


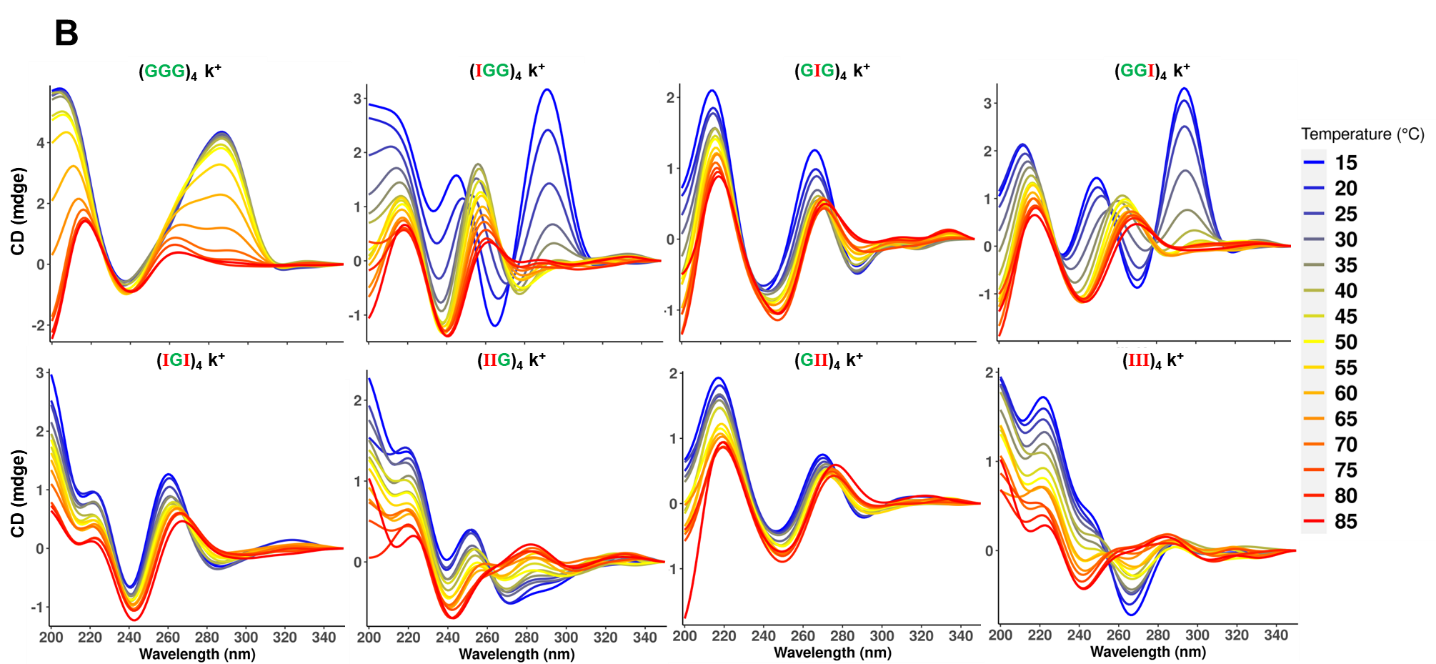


**S5:** A classical representation of the CD melting spectra for all DNAs tested in 100mM Na^+^ (**A**) and in 100 mM K^+^ (**B**). Temperature gradient ranging from 15 ºC (blue) to 85 ºC (red) corresponding with color intensity association with cold/blue and hot/red.


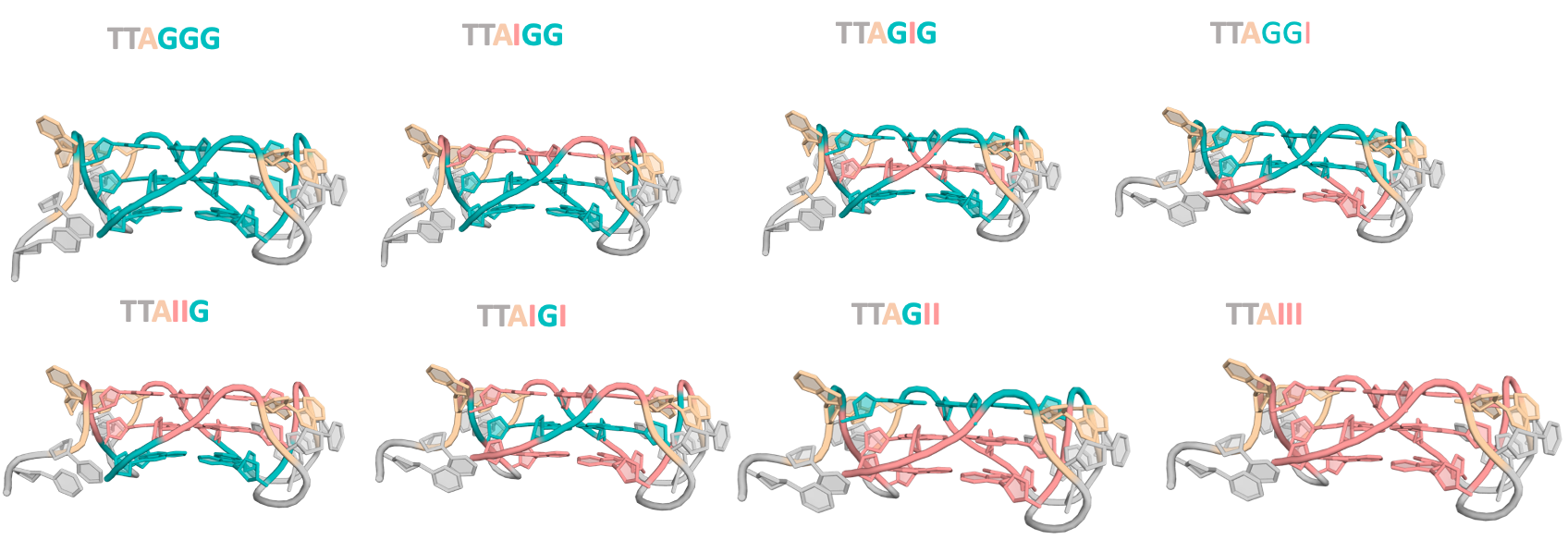


**S6:** 3D structure models of the native and inosine substituted telomeric repeat sequences (TTAGGG) in parallel topology. Only G4 and I4 quartets are possible.


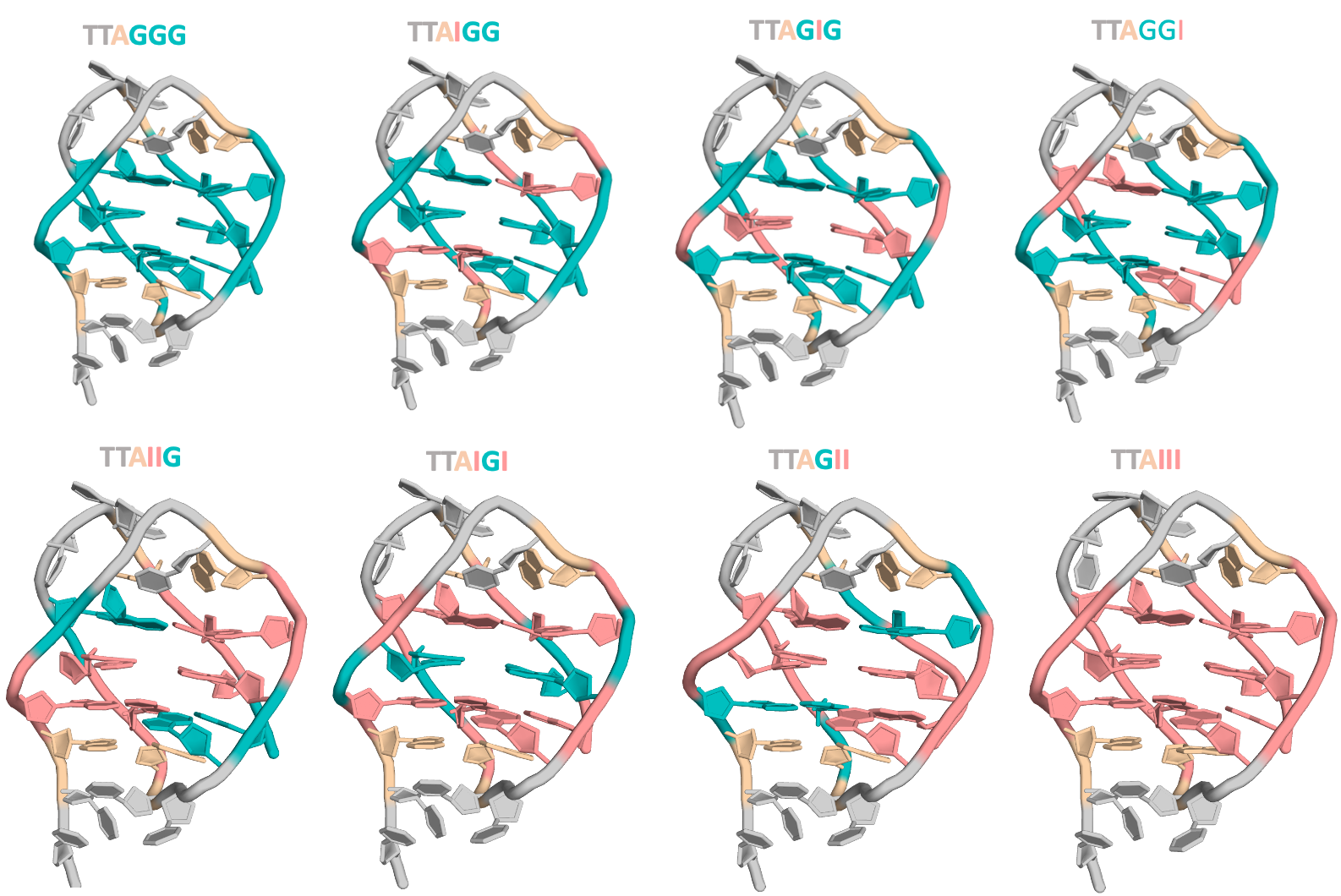


**S7:** 3D structure models of the native and inosine substituted telomeric repeat sequences (TTAGGG) in antiparallel topology. G4, G2-I2, I4 quartets are possible.


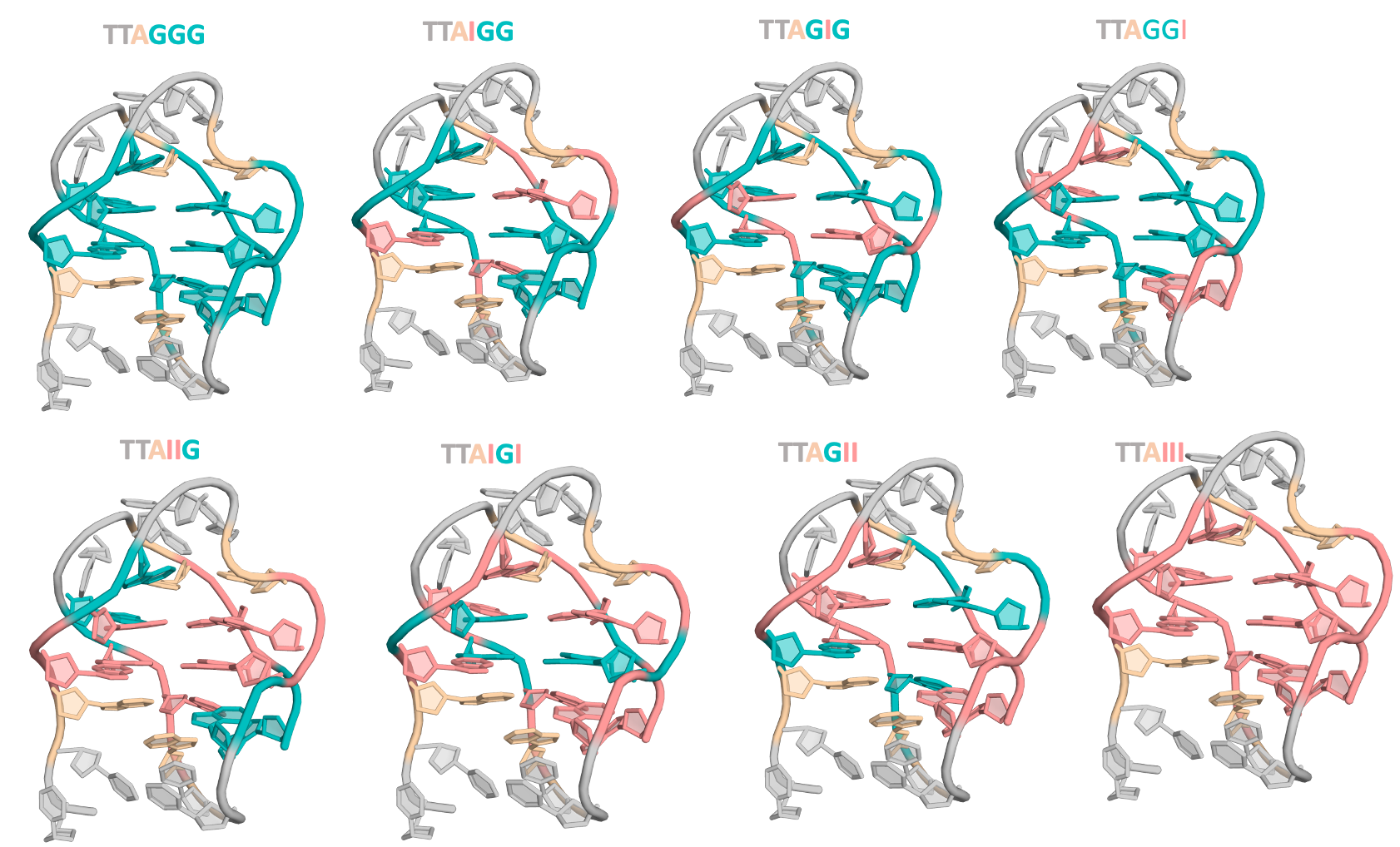


**S8:** 3D structure models of the native and inosine substituted telomeric repeat sequences (TTAGGG) in hybrid topology. G4, I1-G3, I3-G1, I2-G2, I4 quartets are all possible.
